# Clinical Holistic Medicine: Avoiding the Freudian Trap of Sexual Transference and Countertransference in Psychodynamic Therapy

**DOI:** 10.1100/tsw.2008.27

**Published:** 2008-04-14

**Authors:** Søren Ventegodt, Isack Kandel, Joav Merrick

**Affiliations:** ^1^ Quality of Life Research Center, Classensgade 11C, 1 sal, DK-2100 Copenhagen O, Denmark; ^2^ Research Clinic for Holistic Medicine, Copenhagen, Denmark; ^3^ Nordic School of Holistic Medicine, Copenhagen, Denmark; ^4^ Scandinavian Foundation for Holistic Medicine, Sandvika, Norway; ^5^ Interuniversity College, Graz, Austria; ^6^ Faculty of Social Sciences, department of Behavioral Sciences, Ariel University Center of Samaria, Ariel, Israel; ^7^ National Institute of Child Health and Human Development, Jerusalem, Israel; ^8^ Office of the Medical Director, Division for Mental Retardation, Ministry of Social Affairs, Jerusalem, Israel; ^9^ Kentucky Children's Hospital, University of Kentucky, Lexington, USA

**Keywords:** sexuality, sexual healing, infantile autoerotism, schizophrenia, spontaneous regression, physical holding, therapeutic touch, clinical holistic medicine, psychodynamic psychotherapy, STPP, CAM, psychoanalysis, sexual transference and countertransference, vaginal acupressure, Freud's trap, Denmark

## Abstract

Sexual transference and countertransference can make therapy slow and inefficient when libidinous gratification becomes more important for both the patient and the therapist than real therapeutic progress. Sexual transference is normal when working with a patient's repressed sexuality, but the therapeutic rule of not touching often hinders the integration of sexual traumas, as this needs physical holding. So the patient is often left with sexual, Oedipal energies projected onto the therapist as an “idealized father” figure. The strong and lasting sexual desire for the therapist without any healing taking place can prolong therapy for many years, as it often does in psychodynamic psychotherapy and psychoanalysis. We call this problem “Freud's Trap”. Freud used intimate bodywork, such as massage, in the beginning of his career, but stopped, presumably for moral and political reasons. In the tradition of psychoanalysis, touch is therefore not allowed. Recent research in clinical holistic medicine (CHM), salutogenesis, and sexual healing has shown that touch and bodywork (an integral part of medicine since Hippocrates) are as important for healing as conversational therapy. CHM allows the patient to regress spontaneously to early sexual and emotional traumas, and to heal the deep wounds on body, soul, and sexual character from arrested psychosexual development. CHM treats sexuality in therapy more as the patient’s internal affair (i.e., energy work) and less as a thing going on between the patient and the therapist (i.e., transference). This accelerates healing, and reduces sexual transference and the need for mourning at the end of therapy.

## INTRODUCTION

There is plenty of literature on the need to work in abstinence, and almost every therapist on the planet agrees with the Hippocratic ethics of avoiding sexual contact with their patient. Sexual transference and countertransference is, therefore, a concern in psychoanalysis and psychotherapy, but there is a scarcity of papers that analyze this mutual libidinous gratification, in spite of the fact that the issue is highly disturbing to so many therapists[1].

Recently, Irvin D. Yalom, the father of "existential psychotherapy"[2], on a visit to Copenhagen, addressed the taboo of sexual feelings in therapy directly by declaring that: "I have been sexually aroused by patients and so have every therapist I know." A participant in this conference and teacher in psychoanalysis was somewhat uncomfortable to admit that he — at the end of a very difficult, almost 10-year long, four-sessions-a-week, analysis with a mentally ill, sexually abused, female patient — had an erection. More than anything else, this event signified to him that the patient had finally successfully healed not only her sexuality, but also her basic existence; however, he still felt uncomfortable to be aroused by an abused patient, especially because he had been positioned as the abuser in the transference earlier in the therapy.

We must face the fact that therapists are human beings with the same sexuality, and the same feelings of shame and guilt, as other people. This means that whenever a man and a woman are together, as in therapy, and they share intimate details, they will be affected sexually[3,4,5,6]. There will always be some internal reaction and some external reaction towards the other, i.e., transference and countertransference of love and sexuality, so sexuality thus ceases to be entirely the internal affair of the patient. Since the days of Hippocrates, the ethical art of therapy has been not to act on these feelings[7].

Any relationship needs an investment of energy to be of any importance and this energy is our life energy, which is very sexual, as Freud noted correctly[8]. When the therapeutic relationship turns sexually rewarding, the therapist must guard his intention at all times and be certain that he intends to help the patient, not to engage in libidinous gratification, however pleasant and however unavoidable.

Unfortunately, the subconscious drive often wins over the mental and spiritual interest of curing the patient, and in this case, therapy often gets stuck. By addressing the sexual healing explicitly and directly, therapy can move on, but if this does not happen, the therapist and the patient are often hopelessly trapped in what we call the "Trap of Freud": the continuous libidinous gratification that will make the patient pay for many sessions with no real progress, and with great, prolonged, and painful mourning in the end.

It is difficult to know when this state has been reached. One sign of this problem might be that the therapist starts to dream and fantasize not only about coitus with the patient, but also about actually marrying him or her; on the other hand, such fantasies might be necessary for the therapy[4,5]. We must admit that therapy in this situation has almost turned into a real "marriage" between the therapist and the patient. There are so many similarities to marriage that the only major difference is the lack of physical acting out.

For Harold Frederic Searles (1918–), the difference between fantasizing about marrying or having sex with the patient and actually doing so is crucial[4]: Without the fantasy, sexual energy is not available for therapy, but without the taboo on acting out, this energy is channeled into the sexual relationship with the therapist, not the therapy; this taboo, however, brings mourning because the desired relationship is sacrificed for the therapy. Searles also worked under the taboo of touch and from our perspective, this unnatural distance to a person you care for is what created the accumulated and stagnant libidinous energy experienced by Searles, and thus the fantasies. In one study, Searles worked for 900 h, on average, with the patients[9].

Sexual transferences and countertransferences are not bad for therapy; brilliant therapists like Sigmund Freud (1856–1939) and Searles believed that transference and countertransference of love and sexuality were necessary for therapeutic progress, but they must be used wisely[3,4]. The need for sexual healing must be acknowledged and the therapy must address this need directly in order for therapy to progress efficiently. In order for healing to take place (according to the theory of holistic healing[7,10], see discussion below), it is necessary to provide the patient with the support and holding necessary for spontaneous regression to their traumas. What most patients need, according to the Hippocratic medical tradition, is physical and mental contact, love, respect and acceptance, honest conversation, and physical intimacy[11,12].

According to some experts, Freud and other psychoanalysts stopped giving physical holding precisely *because* it encouraged sexual gratification, and this could have been a way to signal to the surrounding world that sexuality was now under control in the therapy. Physical contact and therapeutic touch had been an integral part of holistic medicine since Hippocrates and Freud was, of course, familiar with this tradition because of his own medical roots and his own initial practice (see below how he treated hysteria very much the same way as the Hippocratic doctors).

So we find it highly unlikely that Freud really believed that stopping bodywork would solve the problem of therapists acting out; probably, Freud, who was a politically cunning developer, intended to modernize the somewhat old-fashioned holistic medicine and take it into a medical practice that could be widely accepted and used by his contemporary fellow therapists. We know from his writings that he often reflected deeply on what could be accepted by the press and contemporary culture, and what could not.

Most interestingly, the energy needed for deep existential healing (salutogenesis[13,14]) is what we would call of a maternal character; if the patient receives a nourishing, female, motherly energy, he or she will often spontaneously regress into and heal from early, infantile, sexual traumas. It is important, though, that the therapist does not exclude the male pole in his contact with the patient because treating the supportive energy as only a maternal energy can lead to a serious denial of sexuality.

In many premodern cultures, the medicine man was a person of "double sex", being able to be both father and mother at the same time. The same idea is prevalent in today's Indian yogis; the famous yogi Sai-Baba's name meaning literally mother-father. The Jungian idea of an inner opposite sex (anima and animus) was an important development in psychoanalysis[6] and many psychoanalysts have believed that Freud's limited ability to help schizophrenic patients was due to his lack of willingness to be the mother. Let us quote Harold F. Searles in one of his fine passages:

> "My impression is that Freud himself clung to this father-transference role in order to avoid facing the anxiety associated with the patient's working through their earlier conflicts in relation to him as a mother in the transference. This is a clue, I think, to why Freud considers schizophrenic patients, in whom the resolution of such conflicts is crucial, to be insusceptible to psycho-analytic therapy." [5, p. 440.]

Most interestingly, the reparenting and the care for spirit, mind, and body at the same time is also what characterized the original Hippocratic character medicine[7]. Many Hippocratic procedures had the purpose of rebalancing sexual energies, especially of female patients who received pelvic massage ([7,15,16], see [17] for a review of the classical literature on the method).

The indication for this treatment was "hysteria" (from Greek Hystera: Uterus), believed to signify a broad range of female mental illnesses. This treatment (also called vaginal acupressure) gives intense physical holding to the female patient's body, including the genitals[15,16], allowing her to regress and heal infantile sexual traumas also related to infantile (auto)erotism (see below). This treatment is thus highly rational from a psychodynamic perspective, in spite of obvious ethical problems[15,16] (see below), which presumably inspired Hippocratic doctors to develop their famous ethics.

## SEXUAL TRANSFERENCE AND COUNTERTRANSFERENCE

Sexuality is ubiquitously present in nature and two sexually sound people will always, to some extent, have some bodily sensations of a sexual nature provoked by each other's body. If we were just animals, sexual interest would be constantly and openly present. Being composite creatures with body, mind, and spirit, and Id, Ego, and Self (soul, higher self), the bodily part of us is constantly interested in sex, which is in many ways sublimated, as Freud ingeniously noted, as much of our natural interest in other people comes from sexuality, but is turned into mental and spiritual interest. Researchers in tantra[18] have noticed, as Freud did, that our mental and spiritual energy basically is transformed sexual energy, and here it is important not to fool oneself: It is still sexual energy, just in a more socially acceptable form.

Having stated these plain and well-known facts, we can take a deeper look at sexual transference and countertransference. There has been a strong taboo against these in psychoanalytic and psychodynamic psychotherapy[1], and from the very beginning they were considered a serious threat to the reputation and practice of psychoanalysis[3, p. 170]. The reason for the taboo is not very surprising because who would send their sick young daughter to a man whose primary interest is to engage his sexual energy in her? So from the very beginning, psychoanalysis has — in spite of Freud always stressing frank honesty as a key value — made very smart cover-ups, especially in the language it has been using. Most people do not realize what the Oedipus conflict is about, and they do not want to know either, for this issue is far too provoking. Most sexual transference seems to be of an Oedipal nature and this was the reason for Freud to develop his seemingly strange Oedipus theory: The nurturing relations between children and parents carry extremely strong, but often unconscious, sexual feelings and this energy very often materializes itself in therapy, when the patient is regressing to an early childhood scenario and projecting father or mother (or both) on the therapist[10]. However, Freud did talk about this mother-infant bond as sexual in a broad sense. Psychoanalysis has had to operate throughout its history with a tension between its highly sexual theories and its wish to be accepted in a repressive culture. It has not always gotten everything right, but it would be oversimplistic to say it has covered up sexuality.

These subliminal or conscious sexual complexes and feelings in psychodynamic therapy and psychoanalysis have been resolved in a simple way. When years of intensive contact in therapy have finished the sexual-energetic process, the natural end is a very intense and prolonged mourning. This whole process often takes years and, during this time, many patients will stick firmly to their symptoms since these supply the patient with a justification for staying in the most intense, intimate, and pleasurable psychosexual contact to another human being they have ever experienced.

Meetings four times a week for years are not unusual – literally thousands of sessions. The obvious lack of progress in therapy is understandable if we acknowledge that our body has priority in our subconscious universe, controlling so much of our behavior. This trap of psychoanalysis must be avoided at all costs as it makes therapy expensive and inefficient.

We know, of course, that some psychoanalysts might find our analysis hard to accept, and from a traditional psychoanalytical position, it is clear that what we have stated above could be seen as a misunderstanding and oversimplification of psychoanalysis on a number of fronts. First, psychoanalysts could argue that the mourning is, in part, at least because of the lack of sexual gratification, not because of the loss of it. In the same way, the incest taboo between parents and their children is what compels them to form sexual relationships outside the family of origin. However, there is a kind of mourning involved in the acknowledgment that although daddy finds the child lovely, he is married to mummy and therefore not available. As many fine statistics have documented[9], it is not correct to say that no one benefits from or needs long-term therapy. Another argument that could meet our position in this paper is that it promotes a "one size fits all" type of philosophy that might not be correct; it might simply be that some types of patients need many years of dialog and verbal therapy, and not bodywork.

Our aim with ongoing research is to develop more effective and fast therapy, and so it might definitely turn out in the end that we have been too optimistic of the methods of CHM and the combination of psychotherapy and bodywork; but for now, we prefer to stick to our optimism, especially as this optimism, in itself, seems to accelerate therapy immensely.

The only way to accelerate the process is to address it directly and consciously, to abort the more or less unconscious, mutual plan of a sexual-energetic long-term "marriage". Actually, it is well known from analytic literature[3,4,5,6] that both therapist and patient have intense and ongoing fantasies of sexual intercourse and marriage, and it is, from our perspective, not a shame or a bad thing, but a biologically and completely natural thing, although still a trap that we definitely must be smart enough to avoid.

The only way to avoid being caught by the subliminal sexual rewards of therapy is to address sexual issues openly, and get the therapy going at a well-defined and high speed. We must talk openly about sexuality, address sexual transferences and countertransferences as soon as they are noticed, keep the focus on the goal of therapy, and avoid being afraid to take the patient into deep regression and earlier sexual traumas by using the holding and support needed for this, including therapeutic touch.

## FREUDIAN ROOTS IN HOLISTIC MEDICINE

Most interestingly, Freud did work rather intensively with therapeutic touch in the beginning of his career, very much in the Hippocratic tradition of holistic medical doctors, but stopped giving physical holding to his patients as he continued to developed psychoanalysis. Lauren Nancarrow Clarke writes ([19], p. 8):

> "Freud[20] used physical, body-to-body touch as one of his therapeutic tools... Freud, in several recorded case studies, performed the necessary leg massage and rolling for his hysterical patients to help alleviate their symptoms (see Fraulein Elisabeth von R.'s case, for example). Although touch is not the primary focus of this study, Freud's use of touch raises interesting thoughts about the 'touch taboo' in psychotherapy (Kimble Wrye, 1998)[21]. Additionally, while this practice has been lost, the creator of psychoanalysis thought that touch was an important part of the healing process. If one of the patients' main modes of communication is through the bodily symptoms, why is this no longer an area of focus for all clinicians working today?"

It is well known from "The Cocaine Papers"[22] that Freud did much of his research into the psyche while on cocaine, which has a well-known tendency to enhance libido (the large need of self-medication for sexual problems might be the reason why cocaine is available on every "black market" on the planet). It might very well be that the continuous use of cocaine is not very compatible with intimate bodywork if you want to avoid acting out, but a much more plausible reason that Freud abandoned bodywork might have been the extremely tense, sexual-political, and moral situation of that time (Freud talked about "highly explosive forces"[3, p. 170] making physical contact with a patient questionable, even for a physician.)

## USE OF BODYWORK

Holistic doctors have used bodywork since the time of Hippocrates. Freud abandoned it, but many therapists after Freud, like Wilhelm Reich (1897–1957)[23], continued to use it and noticed that bodywork really was extremely effective in healing, by sending patients back into earlier sexual traumas, including unsolved issues relating to infantile sexuality. Unfortunately, Reich did his therapy in a way that, in spite of it being scientific (and also traditional[17]), was seen as a threat to the therapists and physicians of his time, namely by direct sexual stimulation of his female patients. This situation led to the dramatic actions of burning his brilliant books with his unique research on human sexuality, and a jail sentence and death due to heart failure while in jail.

In Denmark, after two sexual revolutions (in the 1960s and in the 1990s) and legalization of both pornography and prostitution, with pornography in every store and almost every TV program package, and with porn stars becoming heroes on national TV, we still have problems with this kind of explicit, manual, sexological therapy. Today we can talk openly about sexuality and we can use bodywork to take patients into regression, but working directly with sexual stimulation of the patient in the sexological clinic is still highly controversial. Direct sexual stimulation of patients with vibrators for clitoral use is coming into use in holistic sexological therapy by alternative therapists, such as the Danish sexologist Pia Struck, who like a dozen other Danish therapists, has been trained in this method by the American "mother of female masturbation", Betty Dodson[24]. Direct sexual stimulation during therapy must be considered a classical tool of holistic medicine[17] and is, therefore, listed as an advanced tool of CHM[25]; its rationale seems to be to induce a sexual opening when the patient's sexuality has been definitely shot down since early childhood. It might be this ancient tradition of holistic, manual sexology that Freud tried to avoid by inducing the taboo of touch.

The bodywork needed for inducing healing, when the patient has strong sexual transference, is not sexual stimulation, but often just simple, therapeutic, accepting touch, which can be done while the patient has their clothes on[18]. More intensive holding can be given with the patient partly undressed or nude[19], and with more therapists and holders[20], without touch becoming sexual. Acceptance through touch[19] and vaginal acupressure, also called Hippocratic pelvic massage after its appearance in the famous Corpus Hippocraticum[7,17], seem to be valuable tools for giving intensive holding and support to the sexually traumatized female patients[6,7], without direct sexual stimulation.

Interestingly, vaginal acupressure is equivalent to the explorative phase of the pelvic examination and should therefore be legal in most countries. However, as a therapist, you must be absolutely certain that a holistic medical procedure is legal in your country and that you have the needed therapeutic competency, ethical training, and supervision before using it.

At the Research Clinic for Holistic Medicine in Copenhagen, we have noticed that sexual issues and severe existential problems after rape and sexual abuse often can be solved in only 10–20 h of holistic therapy if sufficient bodywork is included, when needed[16,28,29]. The extreme acceleration of therapy from 2,000 h of psychoanalysis (1 h, four times a week, for 10 years) to 10 or 20 h of scientific holistic therapy has been the main reason to include therapeutic touch[18] in our development of CHM[20,31,32].

As a therapist, if one dares to go all the way to working with direct sexual stimulation in the holistic, sexological clinic, even the most severe and chronic sexual problems and dysfunctions can be solved. An example of this is the treatment of anorgasmia, where even in the most difficult cases, lack of orgasm and desire lasting for decades could be solved after only 15 h of intensive therapy[24]. Struck et al. (unpublished paper) found that 93% of 500 patients with anorgasmia were cured in this way and the method had no negative adverse effects.

Unfortunately, not many therapists would like to work so directly with the sexuality and genitals of their patient, as it is possible to do with the most radical advanced tools of holistic, sexological, manual therapy. However, in most cases, simple therapeutic touch will do the job. We must strongly recommend that therapists acknowledge the value of manual therapy and the need for physical holding because many problems come from our childhood and a condition where we did not get sufficient love and care from our parents. When we spontaneously go back to these days of early childhood in therapy, we simply need physical holding – as we did then[7,10].

Psychoanalysts who defend the taboo of touch have disputed the need for physical holding. It has been a constant experience from many therapists now, working on hundreds of patients with many different diseases, that touch is often needed for a complete healing of childhood traumas[33,34,35]. The reason that therapeutic touch is needed seems to be the way information is transferred from body to body, by direct transference of biological information[36,37,38,39,40,41,42,43,44,45]. Especially when the patient has been sexually abused does touch seem to be the key to healing[33,34,35].

## USING SEXUAL ENERGY IN HEALING – THE NOBLE ART OF INTENT

Harold Searles wrote in his excellent paper on "Sexual processes in schizophrenia"[5, p. 441]:

> "This vignette brings up the point, too, that as the patient and therapist encounter prolonged periods of mutual despair at ever resolving the illness, both experience powerful urges to give up the difficult struggle towards a genuinely psychotherapeutic goal, and to settle for a much more primitive goal of finding sexual satisfaction in one another."

Here we see the conflict in the therapy. On the one hand, the mutual sexual interest is what sets the patient free energetically and consciously, and motivates the often-painful exploration into a wounded existence. On the other hand, the same mutual sexual interest can be fixating the therapy until it breaks down in mutual despair and reveals its true sexual nature. Searles continues[5, p. 441]:

> "One may see this phenomenon when mutually gratifying investigative work is interrupted, for long weeks and even months, by a recrudescence of the patient's defensive withdrawal. The therapist, having tested the pleasure of carrying on a relatively high order of collaborative therapeutic investigation with the difficult patient, now has a reason to feel that such gratifications are irretrievably gone, and he apt to be preoccupied more than usual by sexual feelings towards, and fantasies about, the withdrawn patient. Such sequences suggest the extent to which the gratifications of psychotherapeutic or psycho-analytic work represent sublimations of libidinal impulses, which break down, for varying periods of time, during such periods of withdrawal… in the relationship between patient and therapist. Just as sexual behaviour by a schizophrenic person may represent his last-ditch attempts to make or maintain contact with outer reality, or with his own inner self… so the therapist sexual feelings towards the withdrawn patient may be, in part, an unconscious effort to bridge the psychological gulf between then, when more highly refined means have failed."

What Searles shares with us here is extremely important: Behind the independent interest of our Id and Ego, we still have the intentions of the self, and if the therapist is conscious of his intentions and constantly intends to serve his patient every second of the therapy — the real challenge of being a therapist — then sexuality might serve a higher and healing purpose. So Searles noticed in himself that his sexual interest in the patient actually was embedded in his good intent for this patient, as is our physical interest in our children when we are good parents. So after all, being a therapist is not that difficult, one must just be like a good parent.

## TRANSFERENCE OR REGRESSION

One cannot avoid sexual transferences in psychodynamic therapy of any kind, but by focusing directly on the triple rehabilitation of body, mind, and spirit (Id, Ego, and Self/soul), one can take the focus from mutual interest here and now — good for confidence and trust, but bad for therapeutic speed — to the crucial rehabilitation of the patient's talents of body, mind, spirit, love, consciousness, and sexuality. Working on these issues seems to be what heals the existence of the patient, i.e., induces salutogenesis.

The therapeutic schools hold somewhat different opinions on regression. According to most contemporary schools and to holistic medicine in general, salutogenesis happens when the patient regresses to the painful moments where striving for survival forced her/him to stretch fundamental existence and reshape personality at its core. We have coined this radical and total human transformation into a more hardcore and survivable version of "juvenile human metamorphosis" and the deep process of existential healing similarly called "adult human metamorphosis"[33,34,35,36,37,38,39,40,41, 42,43,44,45]. These states are so painful that only the most intensive holding can give sufficient support and this often takes all the intimacy the patient can get. These processes can be extremely resource demanding if the patient is severely traumatized, i.e., by repeat rape or sexual abuse in childhood, and they are best done in a group setting[29]. The "healing crisis" that the patient enters is well described as "holy madness" and the therapist is well advised coming from "crazy wisdom"[46].

Interestingly, when holistic therapy is done with a strong intentional focus on love, consciousness, and sexuality, transference is mostly prevented and the healing process is focused internally in the patient. In the process of salutogenesis[7,13,14,47,48,49,50,51,52], not only the mind heals[53], but also the body[54], sexuality[28], and life as a whole[55,56].

So by working on body, mind, and spirit at the same time, much human suffering can be alleviated and, most interestingly, even the ability to go to work is given back to the patient. Scientific holistic therapy, therefore, also helps the patient's economy, which should be very much appreciated by poor patients and equally by the states that offer free health services to their citizens. To return the patient to society, initiating a process that turns the patient in the end into a valuable person for himself and others and for society at large is the finest goal of therapy, and the only goal that really serves the purpose of rehabilitating the patient's character[7] and by that also his sense of coherence[13,14] and purpose of life[57].

## FETAL SEXUALITY AND INFANTILE AUTOEROTISM

From research in the tradition of tantra[18], we know that sexual health is associated with the ability to contain a large amount of sexual energy. We also know that the ability to control the letting go and acting out of this accumulated sexual energy is essential to sexual health. Problems with containing sexual energy are often experienced as a tension or a pressure, leading to emotional lability, premature ejaculations, frigidity, and many other problems related to sexuality and personality[2,3,6,7,8,18,20].

Most interestingly, the therapeutic regression into infantile sexuality heals our ability to contain huge amounts of sexual energy. The regression into early childhood and into the womb as a fetus is often an extremely sexual experience, but the sexual energies are internally circulated, not circulated between self and other persons, as in mature sexuality. Freud called this "infantile autoerotism", and believed that schizophrenics were ill because they were stuck at this level of psychosexual development (Freud[5, p. 429]), very much in accordance with our own observations from deep therapeutic regression of such patients.

It seems that only if our inner sexual energy system is well functioning and healthy can our body and mind can be healthy. It seems that early traumas that arrest psychosexual development at this stage are causing many of the mental, existential, sexual, and even physical problems that we see in the clinic. It therefore seems necessary that for existential healing (salutogenesis) to take place, the therapeutic work must include early regression and healing of the traumas related to infantile autoerotism.

## ETHICAL ASPECTS

As so often happens in our lives, the rule is: What we most desperately try to avoid will be our destiny. This simply follows from the way our minds work. Everything we hold on to with the mind will subconsciously direct our behavior; also when we cling to something negative. All therapy is about telling the patient to let go of the "mind clinging".

From the very beginning, psychoanalysis has desperately avoided sexual exploitation of patients. This has been regulated by firm rules of not touching and avoiding the physical acting out of sexual transference and countertransference. Sexual interest is, however, not going away because of such rules, and the rules most obviously do not prevent sex between therapist and patient, because this continues to be a huge problem and the largest taboo among physicians and psychotherapists, whether they are classical psychoanalysts, gestalt therapists, or CAM healers.

We believe that there should be firm ethical rules in therapy, but the avoidance of touch has destroyed the therapeutic progress. Touch is a basic human need all the way through life and in all kinds of care[58,59]. Positive, accepting, pleasurable touch is most definitely needed for normal childhood development and, therefore, most definitely needed as the most important part of the holding when the patient regresses to childhood in order to solve traumatic childhood issues.

The healing of sexual traumas needs (more than healing of any other trauma) physical support and therapeutic touch. By avoiding touch in therapy in order to avoid sexual abuse, we believe that Freud and other psychoanalysts ended up with many patients being "married" to their therapist in a "sexually gratifying relationship" of little therapeutic value. The ethical problems connected with Freud's Trap are:

- The patient is deprived of her healing, believing it is on its way, instead of a healing that could happen in less hours of intensive therapy involving physical touch. Therapy without touch can also be fast if you address the issues directly, as done in short-term psychodynamic psychotherapy, where even severe psychiatric illnesses often can be alleviated in 20–40 h[60,61,62] and CHM[53].
- The patient will be deeply involved mentally, emotionally, sexually, and existentially with a therapist for many years, often having her therapist as the closest person in her life, with him being the object of her longings, sexual fantasies, and desires. This energetic "marriage" will deprive her of the possibility of getting the male she really needs and the sexual satisfaction she so desperately longs for. So the patient is basically wasting her life.
- Another important aspect is the question of possible financial exploitation. Independent of Freud's Trap, the use of relative inefficient therapeutic methods will always be unethical for the reasons of prolonging therapy and taking too much money from the patient. The patient eternally trapped in Freud's Trap will be caught like a mouse in a mouse trap; driven by her emotions and desires, projecting her inner male onto the therapist as the "divine" idealized father (or mother if a patient is having a female therapist). She will gratefully and without hesitation spend all her available money on therapy, continuing for many years because it is just such an honor to be with the therapist for 2 or 4 h a week — and such an Oedipal pleasure to finally be "married to Dad". If there is no therapeutic gain and the purpose for meeting is mutual libidinous gratification, this is very much like prostitution, with the therapist being the prostituted "expert lover". But this is not prostitution, as the therapist is not admitting — and often not even aware of — the simple, sexualized purpose of their time together. The therapist experience to work seriously; but she is just a very hard case so solve. The harder the patient's case is, the more desperately will the patient need the therapists help. This necessity of prolonged therapy is not only obvious for the patient and the therapist, but often the patient's whole social network is backing the continuation of the therapy up as extremely and vitally important; everybody is happy that the patient finally found such a brilliant doctor who really gets the therapy going. Seen by a cynical, analytic eye, the patient sitting in Freud's Trap is caught and exploited financially; as the sexual pleasure is mutual it would not be correct to say that she is exploited sexually.
- At the end of therapy, there will be mourning and grief. Therapist and patient must separate because the energy is leaving the relationship as it always will in a sexual relationship without fundamental renewing. So the joy of therapy is converted into the pain of therapy. Much of the pleasure the patient paid for must be returned in the end, without the money being returned.

To summarize, the concept of Freud's Trap gives us a view into a part of psychodynamic therapy and psychoanalysis that is not working well. We find the reason for this to be the taboo of touch. There are many reasons for contemporary therapists not to want to touch their patients; there are restrictive therapeutic rules, there are strategies to avoid being tempted sexually, and strategies for avoiding being accused of sexual abuse. Whatever the reason for not touching, the therapist ends up involving the patient deeply, emotionally, and sexually in a relationship that is supposed to be healing, but because of the taboo of touch, it is not.

Such a relationship is neither truly sexually rewarding nor healing, in spite of an often-strong focus on sexuality and some sexual gratification. The therapist with the patient who is "stuck" ends up very much like a prostitute, with the client coming to the "expert lover" for love sessions, but the costumers who are buying this kind of therapy are paying for something that neither develops into the real sex the patient is longing for, nor into the healing she actually pays for. We think that the taboo of touch is a historical mistake that prolongs therapy for years; we do not find that Freud's Trap causes any direct harm to the patients.

We acknowledge that psychoanalysts might disagree with our position in this paper. We admit that we come from the old tradition of Hippocratic holistic medicine, where touch has been an integral part of medicine for millennia, and this gives us very different experiences than those of psychoanalysis and psychodynamic psychotherapy that work with all the pragmatic restrictions of the taboo of touch.

## CONCLUSIONS

Sexual transference and countertransference have been large and unsolved problems in psychoanalysis and psychodynamic psychotherapy as they often make therapy slow and inefficient because of mutual libidinous gratification of the therapeutic relationship subconsciously being more important than therapeutic progress. Purposeful and expressive work on healing the patient’s sexuality using bodywork often takes the patient spontaneously into deep regression, all the way back to sexual traumas in early childhood and even into the womb.

Holistic doctors have used a combination of conversational therapy and bodywork ever since the time of Hippocrates. Freud also used intimate bodywork, such as massage, in the beginning of his career, but stopped, presumably for moral and political reasons. In the classical tradition of psychoanalysis and psychodynamic psychotherapy, touch is not allowed, especially not when related to the patient’s sexuality and genitals.

CHM integrates, in the classical tradition of Hippocratic holistic medicine, psychodynamic psychotherapy and therapeutic touch, making it possible to support the healing of the patient’s sexuality on the physical level. Recent research in holistic medicine, salutogenesis, and sexual healing has shown that touch and bodywork are as important for healing as conversational therapy[63,64].

CHM has also shown good results, presumably because it integrates psychodynamic psychotherapy and therapeutic touch[7,10,26,65]. It allows the patient to regress spontaneously to early sexual and emotional traumas, and heal the deep wounds on body, soul, and sexual character from arrested psychosexual development. CHM treats sexuality in therapy more as the patient’s internal affair (i.e., energy work) and less as a thing going on between the patient and the therapist (i.e., transference). This form dramatically accelerates healing and reduces intensity of the sexual (Oedipal) bonding between therapist and patient and, as a consequence, the experience of loss and need for mourning at the end of therapy.

## ACKNOWLEDGMENTS

The Danish Quality of Life Survey and the Quality of Life Research Center was, from 1991–2005, supported by grants from the 1991 Pharmacy Foundation, the Goodwill-fonden, the JL-Foundation, E. Danielsen and Wife's Foundation, Emmerick Meyer's Trust, the Frimodt-Heineken Foundation, the Hede Nielsen Family Foundation, Petrus Andersens Fond, Wholesaler C.P. Frederiksens Study Trust, Else & Mogens Wedell-Wedellsborg's Foundation, and IMK Almene Fond. The research was approved by the Copenhagen Scientific Ethical Committee under number (KF)V.100.2123/91 and later correspondence.
